# Author Correction: Human FCHO1 deficiency reveals role for clathrin-mediated endocytosis in development and function of T cells

**DOI:** 10.1038/s41467-020-15946-x

**Published:** 2020-04-20

**Authors:** Marcin Łyszkiewicz, Natalia Ziętara, Laura Frey, Ulrich Pannicke, Marcel Stern, Yanshan Liu, Yanxin Fan, Jacek Puchałka, Sebastian Hollizeck, Ido Somekh, Meino Rohlfs, Tuğba Yilmaz, Ekrem Ünal, Musa Karakukcu, Türkan Patiroğlu, Christina Kellerer, Ebru Karasu, Karl-Walter Sykora, Atar Lev, Amos Simon, Raz Somech, Joachim Roesler, Manfred Hoenig, Oliver T. Keppler, Klaus Schwarz, Christoph Klein

**Affiliations:** 1 Department of Pediatrics, Dr. von Hauner Children’s Hospital, University Hospital, LMU, Munich, Germany; 2 Institute for Transfusion Medicine, University of Ulm, Ulm, Germany; 3 Max von Pettenkofer Institute, Virology, National Reference Center for Retroviruses, Faculty of Medicine, LMU München, Munich, Germany; 4 Department of Pediatrics, Division of Pediatric Hematology & Oncology, Erciyes University, Kayseri, Turkey; 5 Department of Pediatrics, Division of Pediatric Immunology, Erciyes University, Kayseri, Turkey; 6 Department of Pediatric Hematology/Oncology, Hannover Medical School, Hannover, Germany; 7 Pediatric Department A and the Immunology Service, Jeffrey Modell Foundation Center, Edmond and Lily Safra Children’s Hospital, Sheba Medical Center, Tel Hashomer and Sackler Faculty of Medicine Tel Aviv University, Tel Aviv, Israel; 8 Department of Pediatrics, Carl Gustav Carus Technical University Dresden, Dresden, Germany; 9 Department of Pediatrics, University Medical Centre Ulm, Ulm, Germany; 10 German Center for Infection Research (DZIF), Partner Site Munich, Munich, Germany; 11 Institute for Clinical Transfusion Medicine and Immunogenetics Ulm, German Red Cross Blood Service Baden-Wuerttemberg, Hessen, Germany; 12 Present Address: Institute for Immunology, Biomedical Center Munich, Ludwig-Maximilians-Universität München, Planegg-Martinsried, 82152 Munich, Germany

**Keywords:** Endocytosis, Disease genetics, T cells, Primary immunodeficiency disorders

Correction to: *Nature Communications* 10.1038/s41467-020-14809-9, published online 25 February 2020.

In the original version of this Article, previous work by Calzoni et al.^51^ was inadvertently misrepresented in the penultimate paragraph of the Discussion. It incorrectly read ‘Very recently, Calzoni *et al*. published a short letter and reported biallelic mutations in *FCHO1* in four families. The phenotypes of these patients resembled the phenotype of our patients, but no functional experiments or proof-of-causality was provided. Based on experiments in activated T-cell blasts, the authors concluded that in the absence of FCHO1, CME is globally affected. In contrast, our data support the concept that FCHO1 does not globally affect CME^51^.’

The correct version states ‘While our study was under review, Calzoni *et al*. reported biallelic mutations in *FCHO1* in four families^51^. The phenotypes of these patients resembled the phenotype of our patients. Based on experiments in activated T-cell blasts, the authors concluded that in the absence of FCHO1, CME of the transferrin receptor is affected^51^. In contrast, our data support the concept that FCHO1 does not control internalization of the transferrin receptor and mediates more subtle effects on CME.’

This has been corrected in both the PDF and HTML versions of the Article.

