# Supplementary material for: Human FCHO1 deficiency reveals role for clathrin-mediated endocytosis in development and function of T cells
Source: Nat Commun. 2020 Feb 25;11:1031. doi: 10.1038/s41467-020-14809-9 (PMC7042371; doi:10.1038/s41467-020-14809-9)
Supplement: Supplementary file 1 — Supplementary Information [file 41467_2020_14809_MOESM1_ESM.pdf]

Supplementary Information

Human FCHO1 deficiency reveals role for clathrin-mediated endocytosis in development and function of T cells

Łyszkiewicz and Ziętara et al.

Sup Figure 1

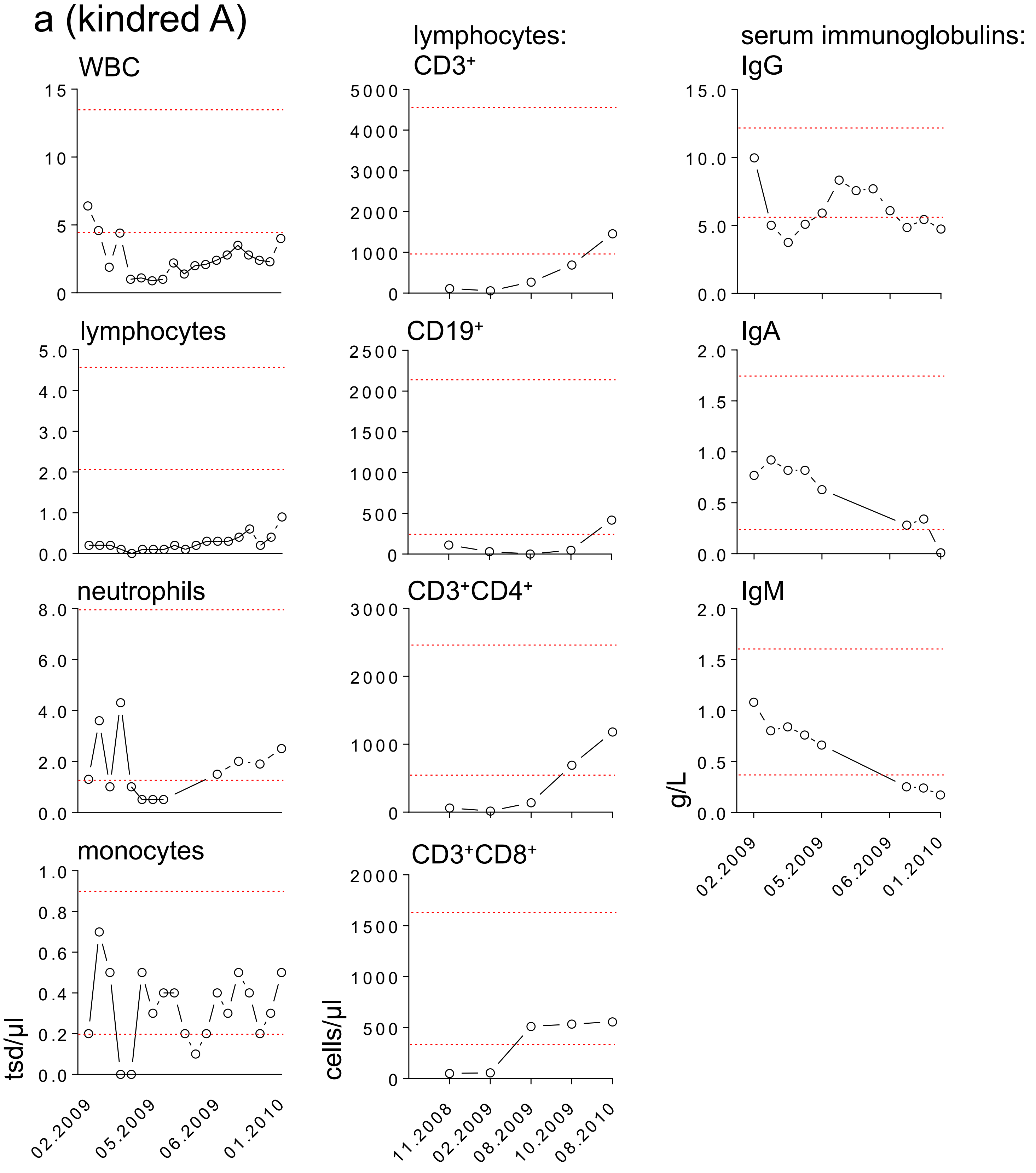
a (kindred A)
WBC
lymphocytes
neutrophils
monocytes
tsd/µl
02.2009
05.2009
06.2009
01.2010
lymphocytes:
CD3+
CD19+
CD3+CD4+
CD3+CD8+
cells/µl
11.2008
02.2009
08.2009
10.2009
08.2010
serum immunoglobulins:
IgG
IgA
IgM
g/L
02.2009
05.2009
06.2009
01.2010

Sup Figure 1

b (kindred B)

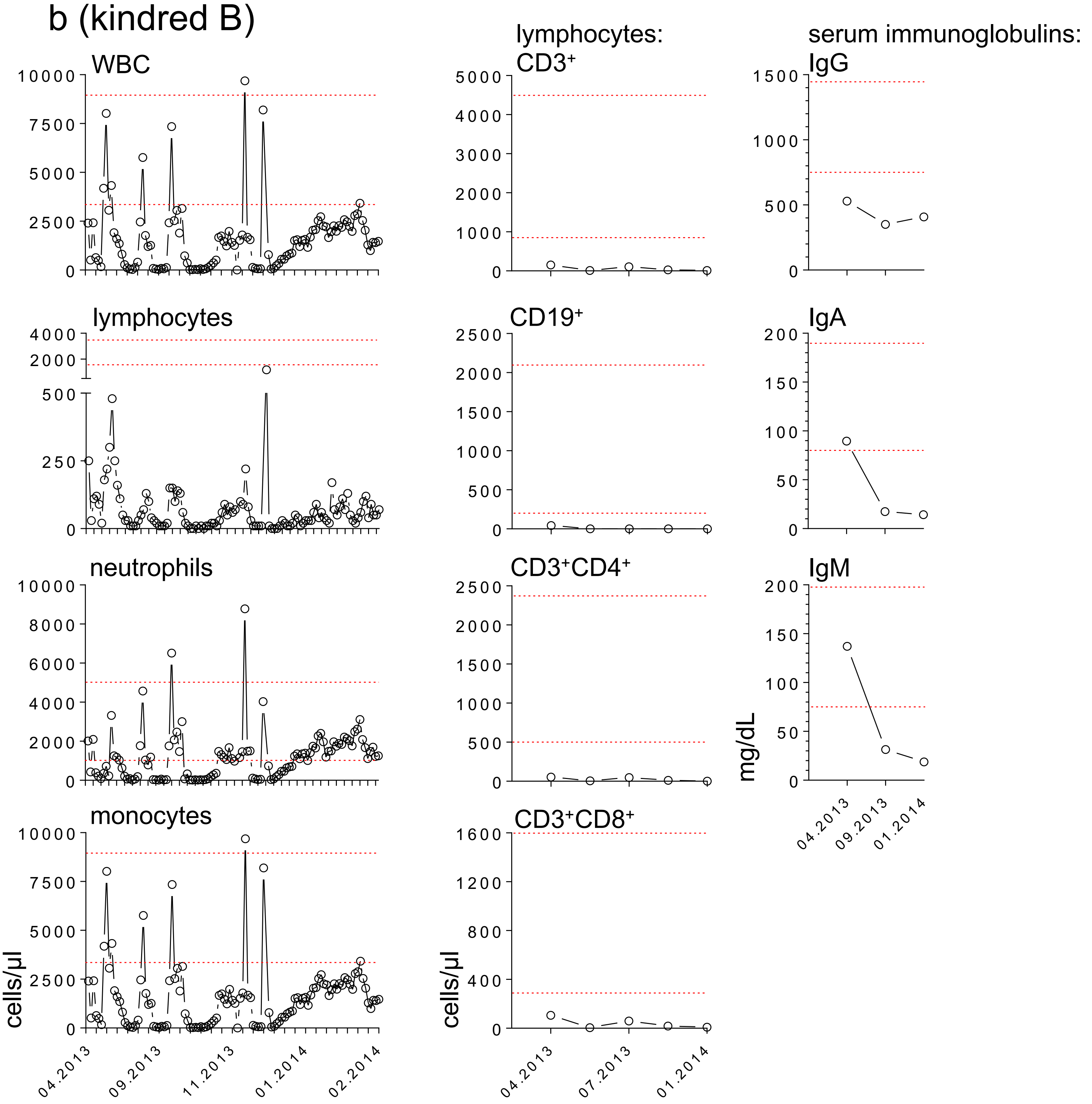

Sup Figure 1

c (kindred C)

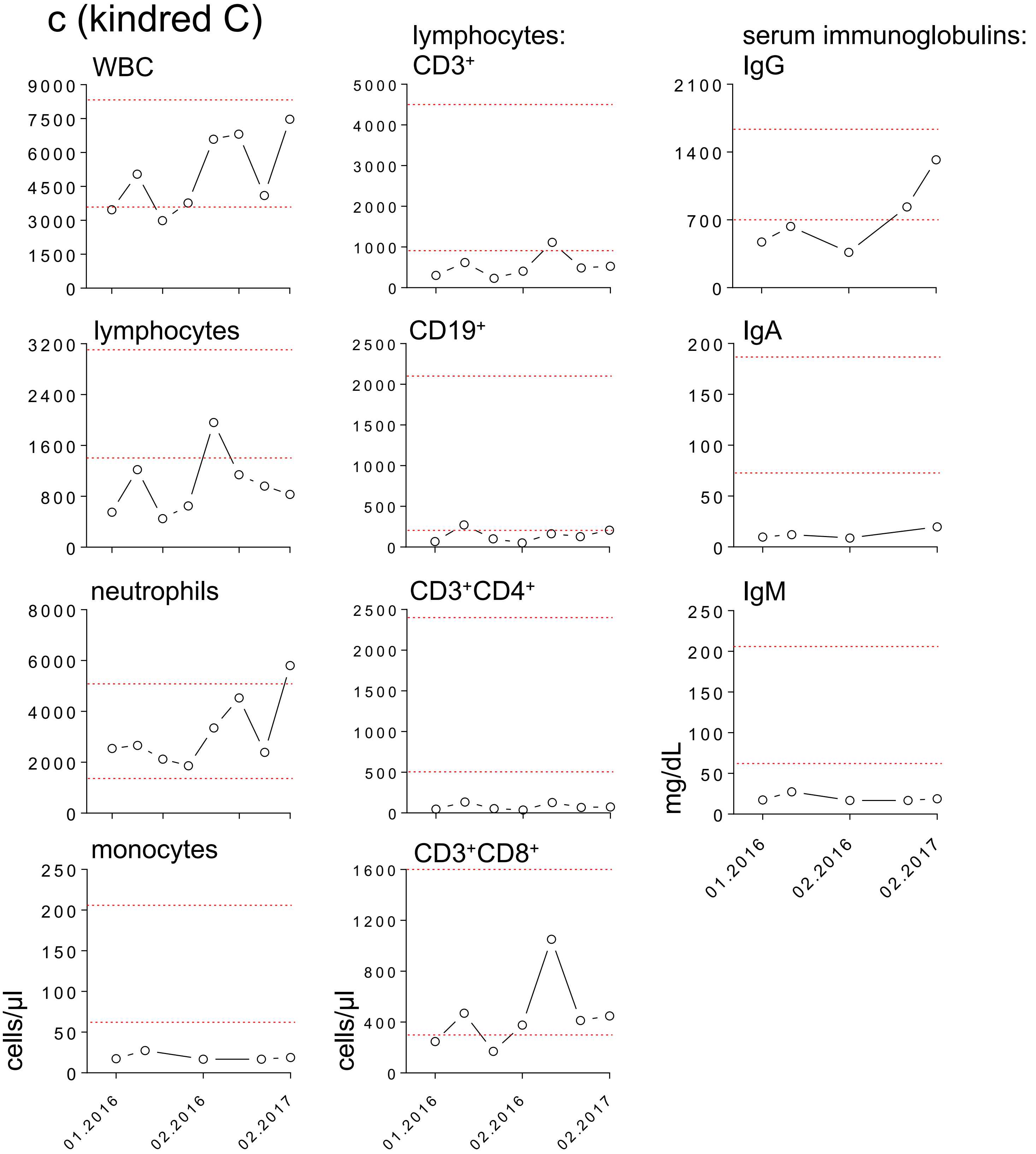

Sup Figure 1
d (kindred D)

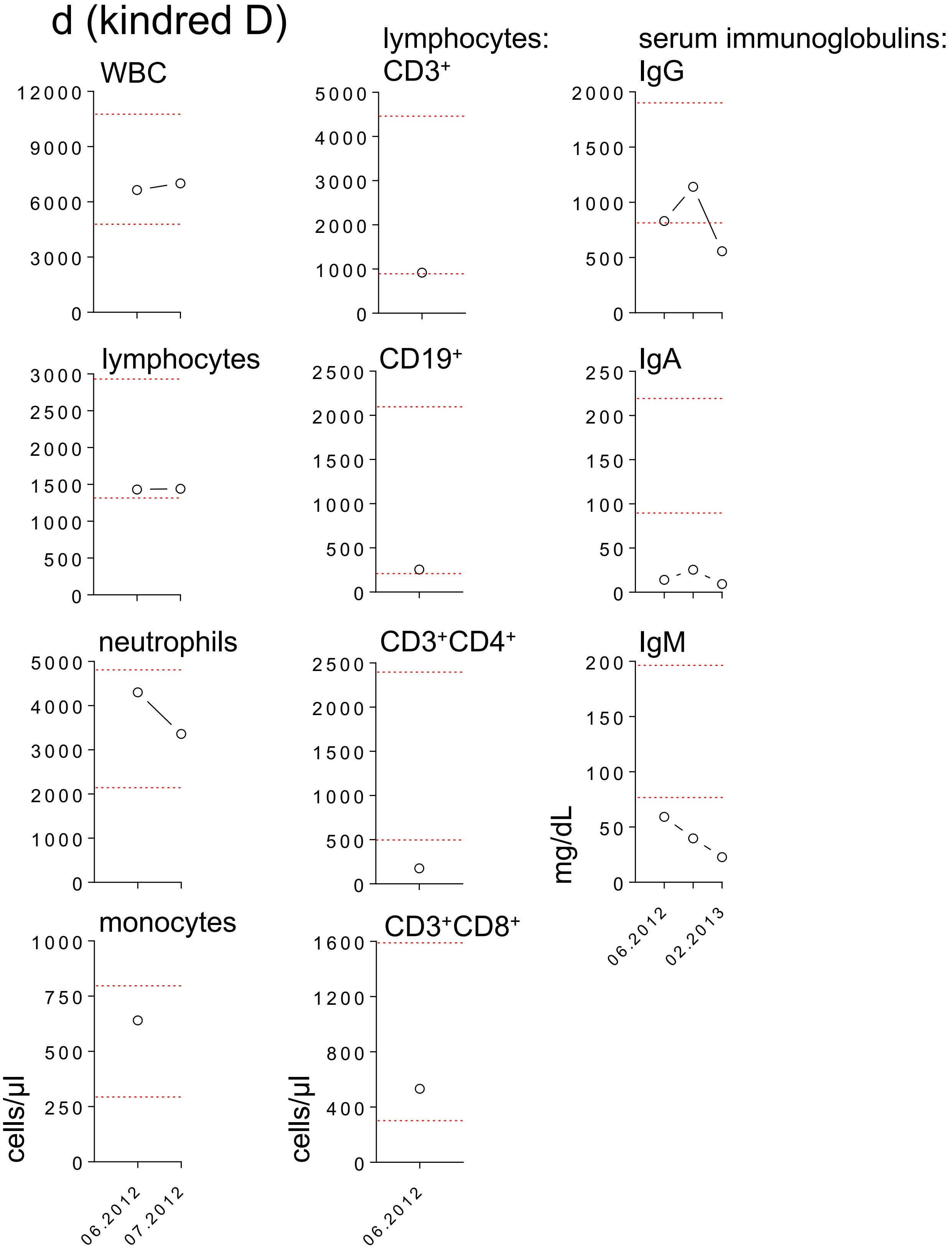

Supplementary Figure 1. SCID-like lymphopenia phenotype in patients carrying homozygous mutations in the *FCHO1* locus. (a-d) Absolute numbers of total white blood cells (WBC) and main populations of leukocytes (left column), main populations of peripheral blood lymphocytes (central column) and serum immunoglobulins (right column) of index cases from kindred A to D. (a) Please note recovery of both T and B lymphocytes upon allogeneic hematopoietic stem cell transplantation (indicated by arrow). The normal range of serum immunoglobulin levels can be attributed to IVIg therapy. (a-d) All presented data were obtained from certified clinical diagnostic laboratories. Red lines on charts show the normal range.

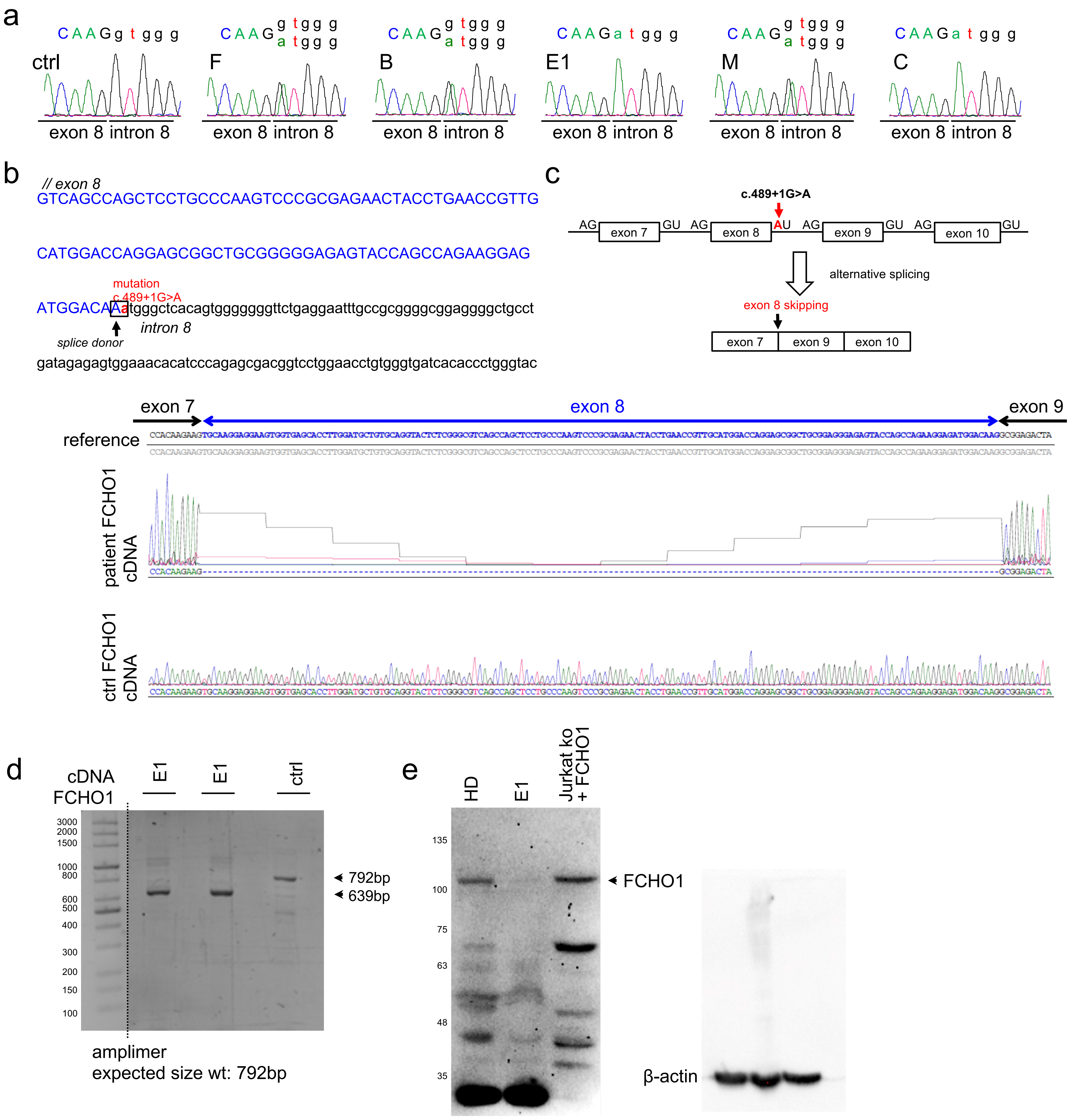

Supplementary Figure 2. Kindred E-associated mutation affects FCHO1 splicing. (a) Sanger sequencing chromatograms indicating a homozygous mutation in exon 7 of index patient c.489+1G>A. (b) A sequence of pre-spliced mRNA with indicated mutation (red). (c) Scheme showing the putative effect of c.489+1G>A mutation on mRNA splicing. Please note skipping of 153 nucleotides of exon 8, resulting in an in-frame protein shortened by 51 aa (6 kDa). (d) FCHO1 cDNA from patient fibroblasts (E1). Wild-type and alternatively spliced cDNA fragments are indicated with arrows. (e) FCHO1 Western blot performed on lysates of a healthy donor and patient fibroblasts.

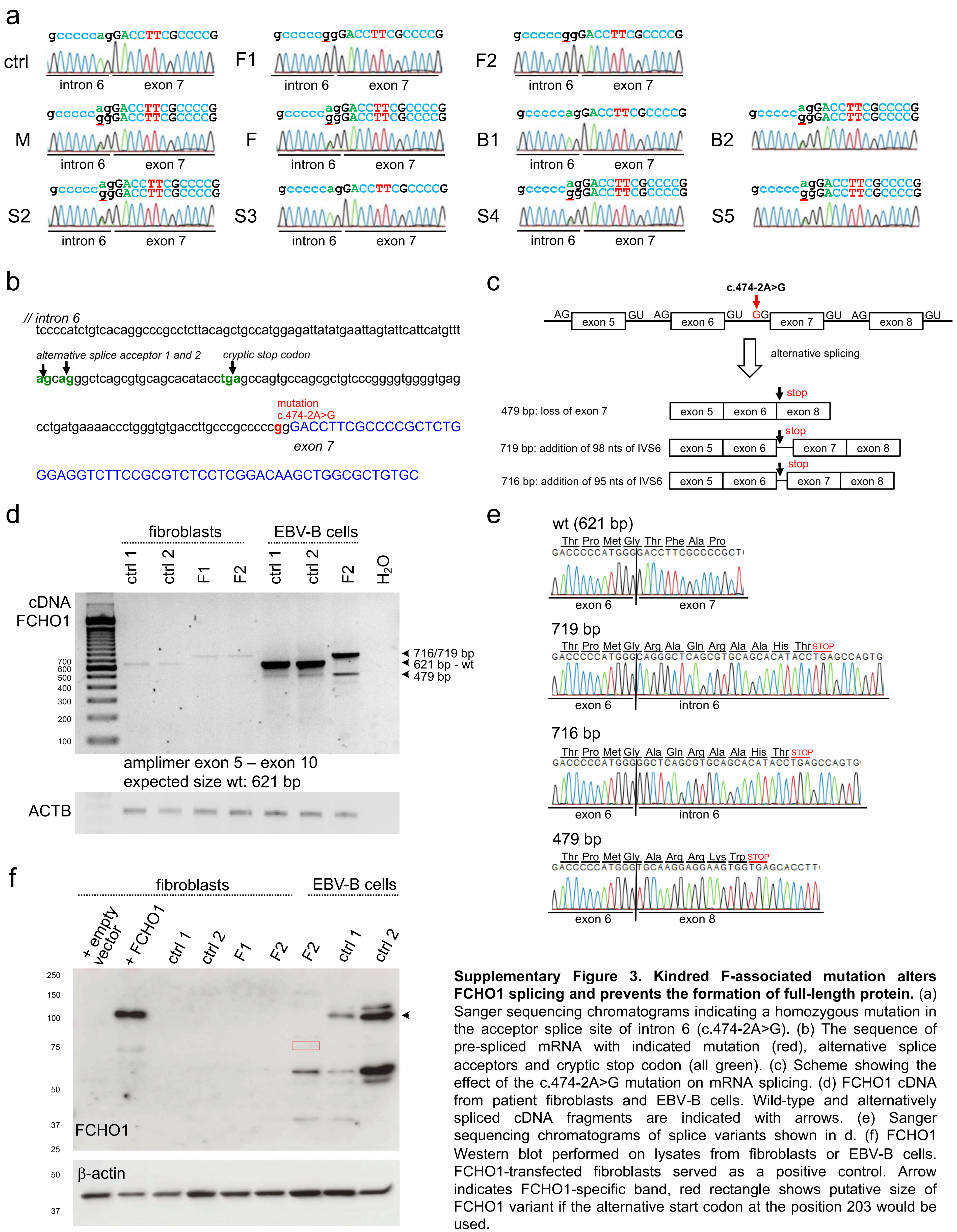

Supplementary Figure 3. Kindred F-associated mutation alters FCHO1 splicing and prevents the formation of full-length protein. (a) Sanger sequencing chromatograms indicating a homozygous mutation in the acceptor splice site of intron 6 (c.474-2A>G). (b) The sequence of pre-spliced mRNA with indicated mutation (red), alternative splice acceptors and cryptic stop codon (all green). (c) Scheme showing the effect of the c.474-2A>G mutation on mRNA splicing. (d) FCHO1 cDNA from patient fibroblasts and EBV-B cells. Wild-type and alternatively spliced cDNA fragments are indicated with arrows. (e) Sanger sequencing chromatograms of splice variants shown in d. (f) FCHO1 Western blot performed on lysates from fibroblasts or EBV-B cells. FCHO1-transfected fibroblasts served as a positive control. Arrow indicates FCHO1-specific band, red rectangle shows putative size of FCHO1 variant if the alternative start codon at the position 203 would be used.

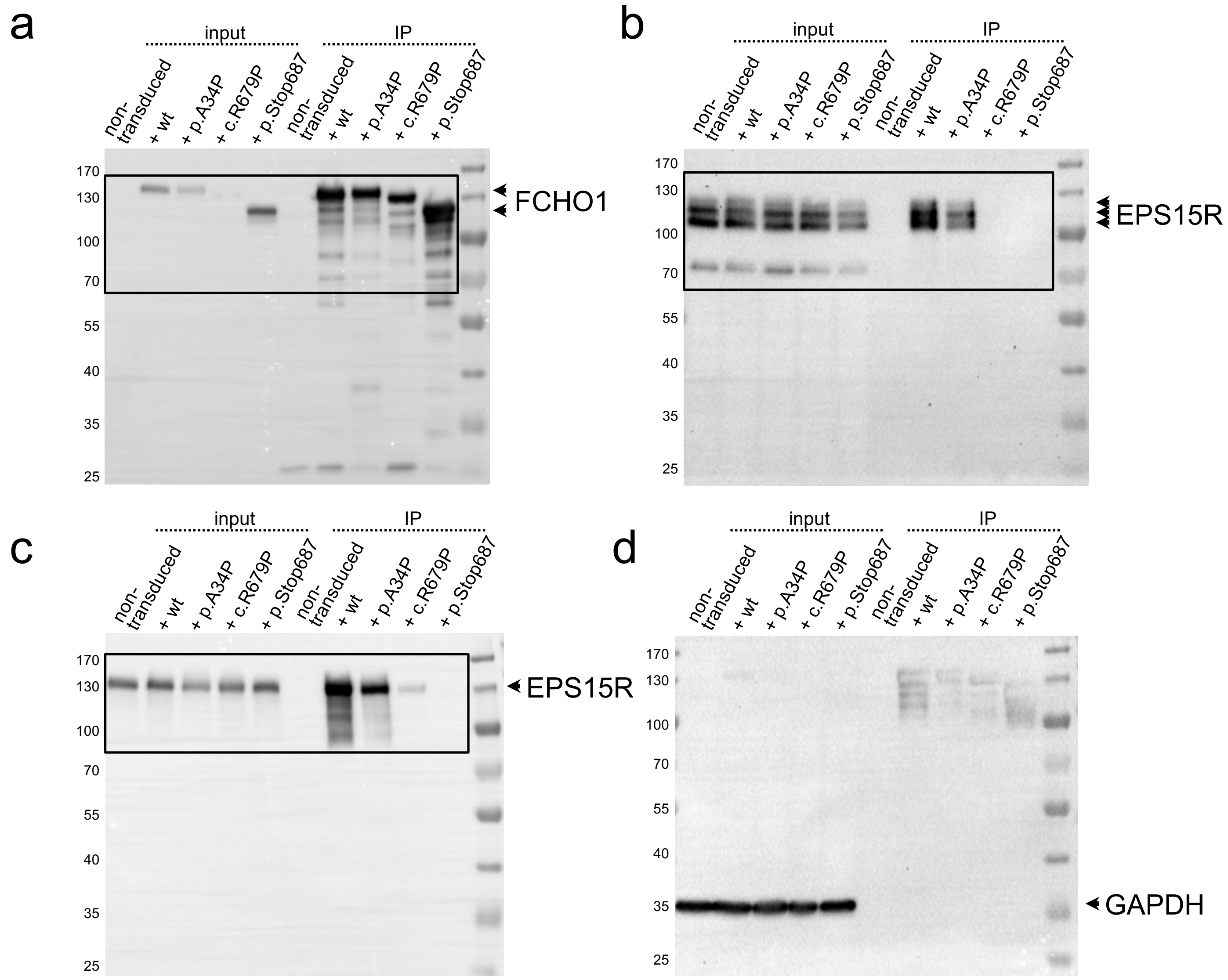

Supplementary Figure 4. Uncropped Western blots images corresponding to data shown in Figure 2. Frames show the fragments displayed in the main figure. The molecular mass of proteins is shown in kDa.

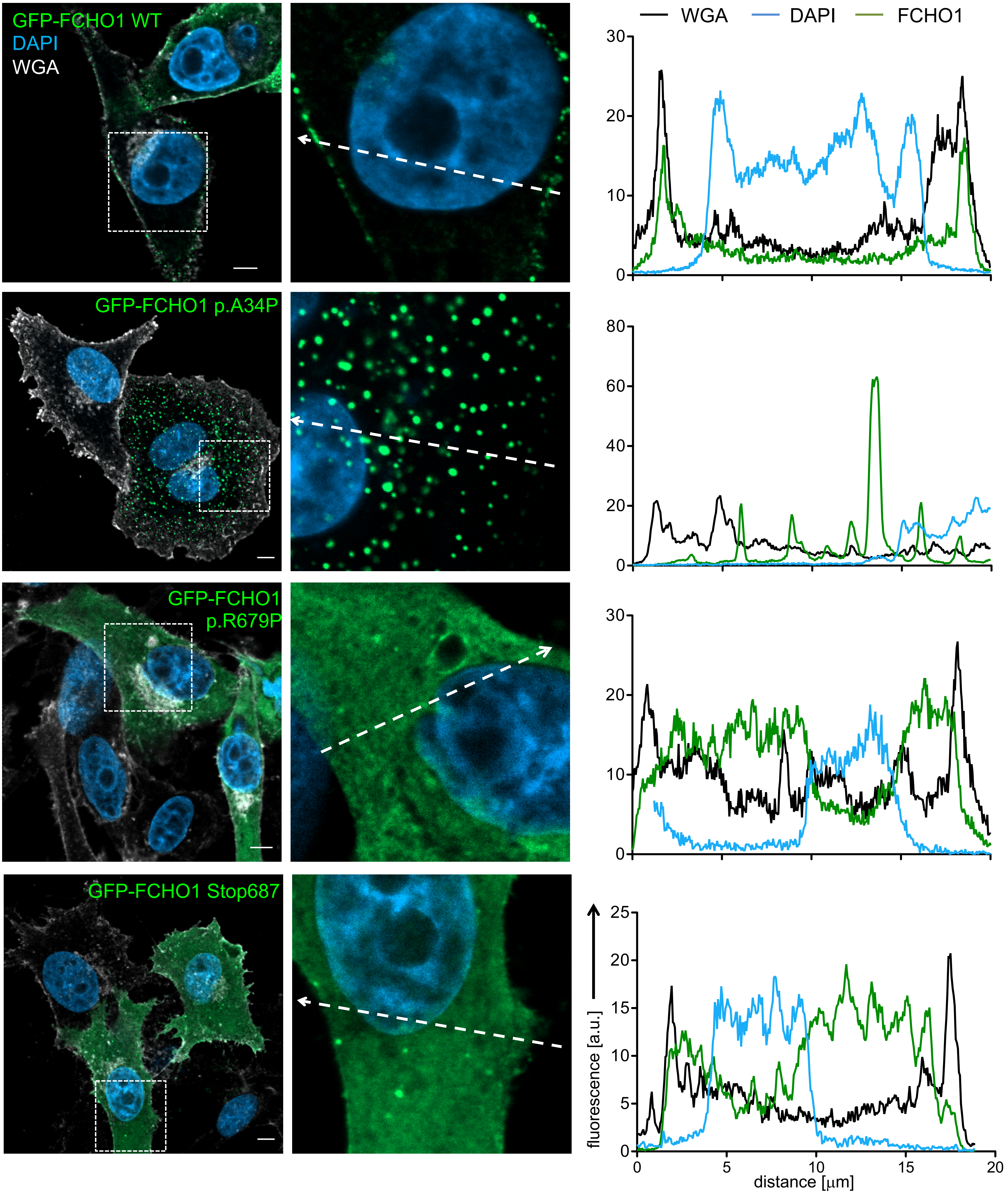

Supplementary Figure 5. Mutations in FCHO1 lead to dissociation of protein from the plasma membrane, yet do not affect overall plasma membrane organization. FCHO1$^{-/-}$ SK-MEL-2 cells were transiently transfected with GFP-fusion protein constructs, carrying indicated mutants of FCHO1 (green on both image and fluorescence plots). Cells were co-stained with WGA (Wheat Germ Agglutinin) conjugated to Alexa Fluor 647 (white on the image, black line on the fluorescence plot) to visualize plasma membrane and DAPI (blue on both image and fluorescence plots) to visualize nucleus. Only wt GFP-FCHO1 almost perfectly correlates with WGA staining of the plasma membrane, whereas both F-BAR domain (pA34P) and µHD domain (p.R679P and Stop687) mutants do not, and the p.A34P mutant is completely dissociated from the plasma membrane (second panel from the top). Data are representative of two independent experiments (two independent transfections). Scale bars 5 µm, enlarged boxes 20 µm, length of the arrows 20 µm.

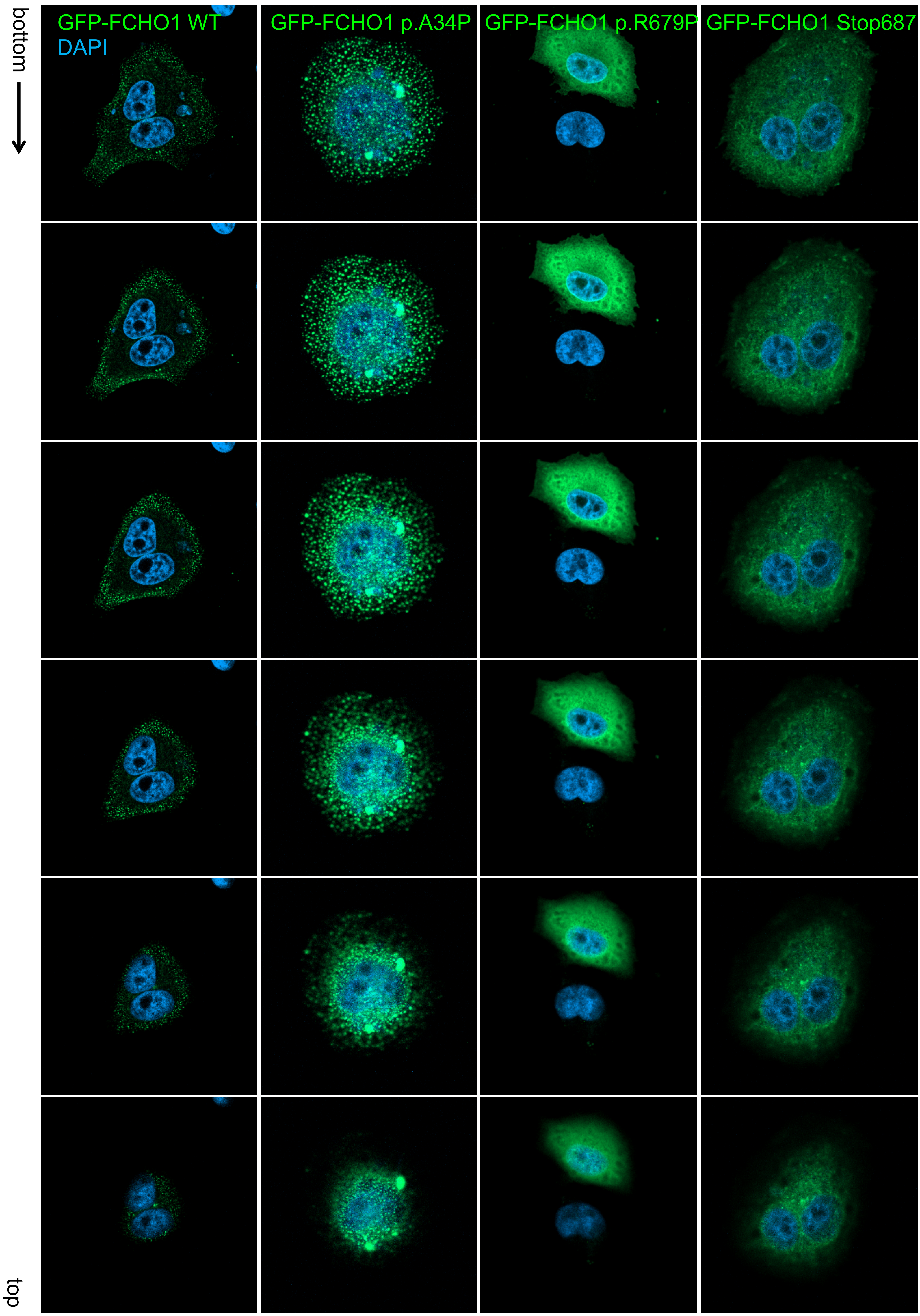

Supplementary Figure 6. Mutations in FCHO1 lead to dssociation of protein from the plasma membrane.
Optical sections (Z-stacks every 40 µm) for 3-D reconstruction and six selected optical sections from whole depth of the cells from the bottom to the top. FCHO1$^{-/-}$ SK-MEL-2 cells were transiently transfected with GFP-fusion protein constructs, carrying indicated mutants of FCHO1. Optical sections visualize dissociation of all FCHO1 mutants from the plasma membrane as compared to wt GFP-FCHO1. Data are representative of two independent biological experiments (two independent transfections).

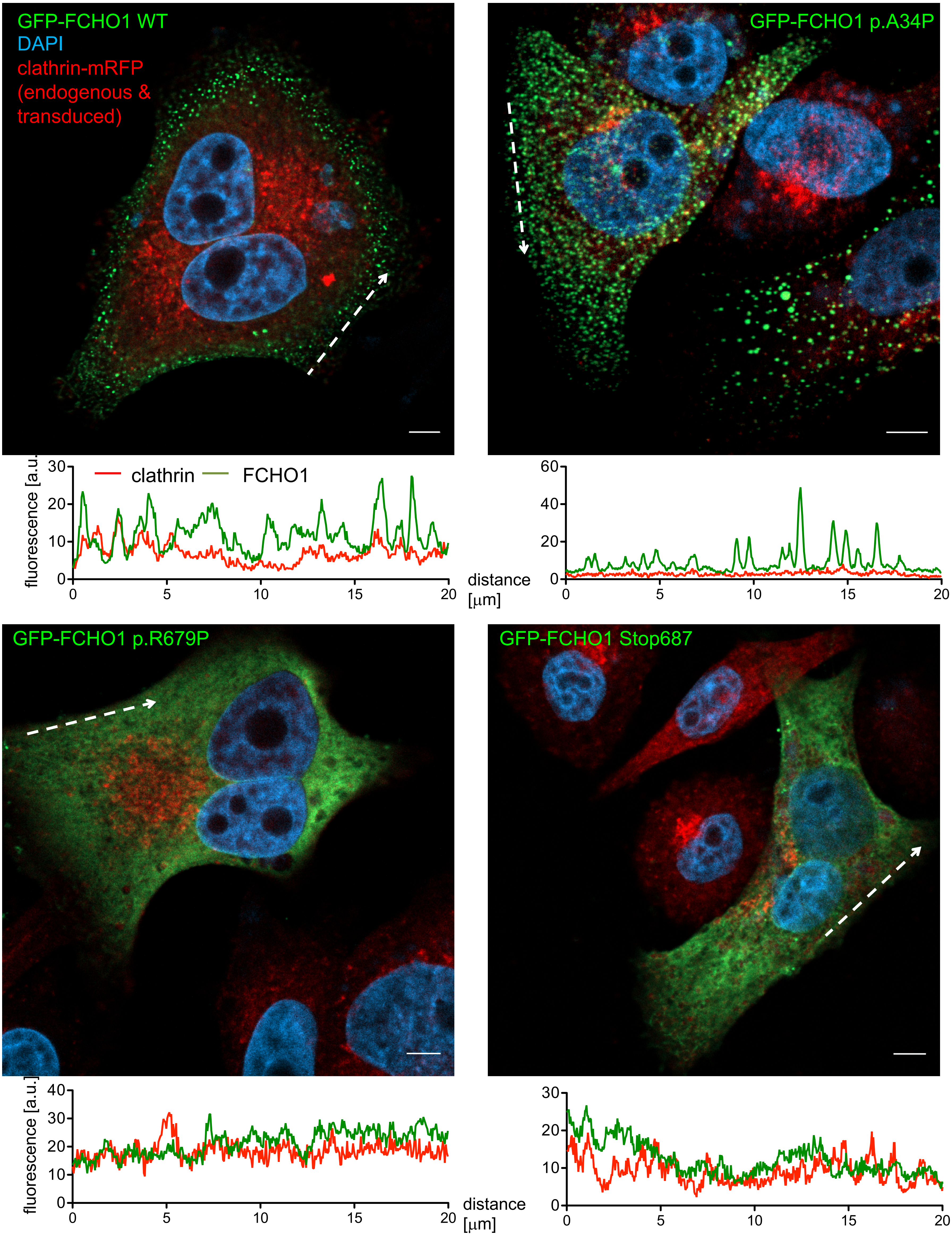

Supplementary Figure 7. FCHO1 mutants fail to colocalize with clathrin. In order to improve clathrin signal-to-noise ratio, FCHO1$^{-/-}$ SK-MEL-2 cells expressing RFP-tagged clathrin light chain from endogenous locus (CLTA$^{RFP/wt}$) were stably transduced with retroviral particles encoding clathrin light chain tagged with mRFP and subsequently transiently transfected with various GFP-FCHO1 plasmids. Representative confocal microscopy pictures show that all mutants but not wt FCHO1 fail to colocalize with clathrin. Arrows indicate regions where both RFP and GFP fluorescence intensities have been displayed on histograms below each image. In contrast to SK-MEL-2 cells engineered to express RFP from a single native locus of endogenous clathrin light chain A (used in all other experiments), additional overexpression of the clathrin-mRFP fusion protein leads to non-physiological accumulation of RFP signal near the nucleus.

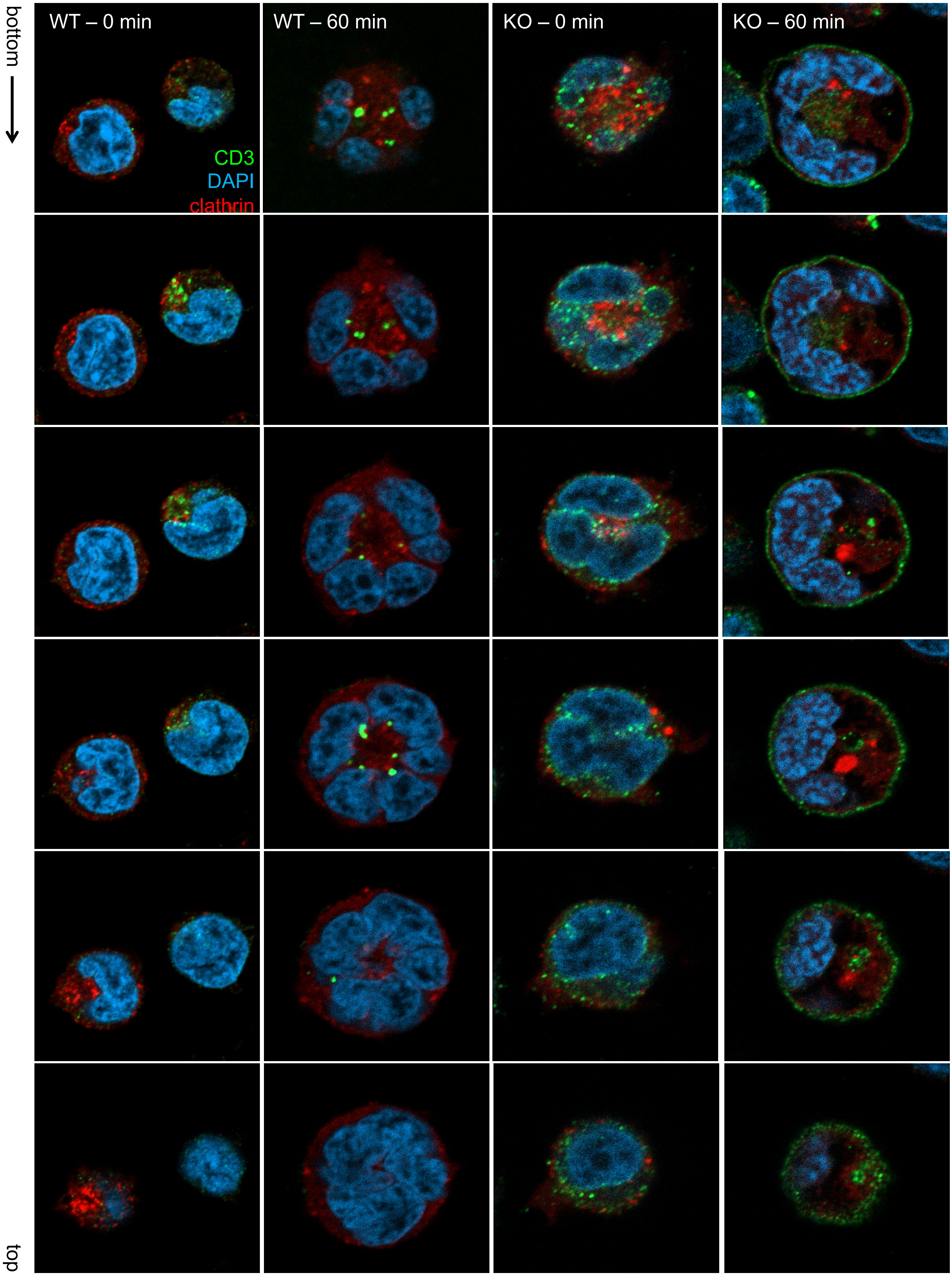

Supplementary Figure 8. This data supplements the 2D data presented on Figure 4a and shows that in the absence of FCHO1 surface CD3 is not efficiently internalised from the plasma membrane. Z-stack for 3-D reconstruction and six selected optical sections from whole length of the cells bottom to the top. Jurkat wt and FCHO1$^{-/-}$ clones were stably transduced with RFP-clathrin and stimulated for 60 min with glass-bound anti-CD3 antibody (OKT-3). It supplements 2D data presented on Figure 4a and shows that in the absence of FCHO1 surface CD3 is not efficiently internalised form the plasma membrane.

a

CD3ε
```
        10         20         30         40         50         60         70         80         90        100        110
MQSGTHWRVL GLCLLSVGVW GQDGNEEMGG ITQTPYKVSI SGTTVILTCP QYPGSEILWQ HNDKNIGGDE DDKNIGSDED HLSLKEFSEL EQSGYYVCYP RGSKPEDANF
       120        130        140        150        160        170        180        190        200
YLYLRARVCE NCMEMDVMSV ATIVIVDICI TGGLLLLVYY WSKNRKAKAK PVTRGAGAGG RQRGQNKERP PPVPNPDYEP IRKGQRDLYS GLNQRRI
                                               cytoplasmic domain                                 DxY sorting motif
```

CD3γ
```
        10         20         30         40         50         60         70         80         90        100        110
MEQGKGLAVL ILAIILLQGT LAQSIKGNHL VKVYDYQEDG SVLLTCDAEA KNITWFKDGK MIGFLTEDKK KWNLGSNAKD PRGMYQCKGS QNKSKPLQVY YRMCQNCIEL
       120        130        140        150        160        170        180
NAATISGFLF AEIVSIFVLA VGVYFIAGQD GVRQSRASDK QTLLPNDQLY QPLKDREDDQ YSHLQGNQLR RN
```

CD3δ
```
        10         20         30         40         50         60         70         80         90        100        110
MEHSTFLSGL VLATLLSQVS PFKIPIEELE DRVFVNCNTS ITWVEGTVGT LLSDITRLDL GKRILDPRGI YRCNGTDIYK DKESTVQVHY RMCQSCVELD PATVAGIIVT
       120        130        140        150        160        170
DVIATLLLAL GVFCFAGHET GRLSGAADTQ ALLRNDQVYQ PLRDRDDAQY SHLGGNWARN K
```

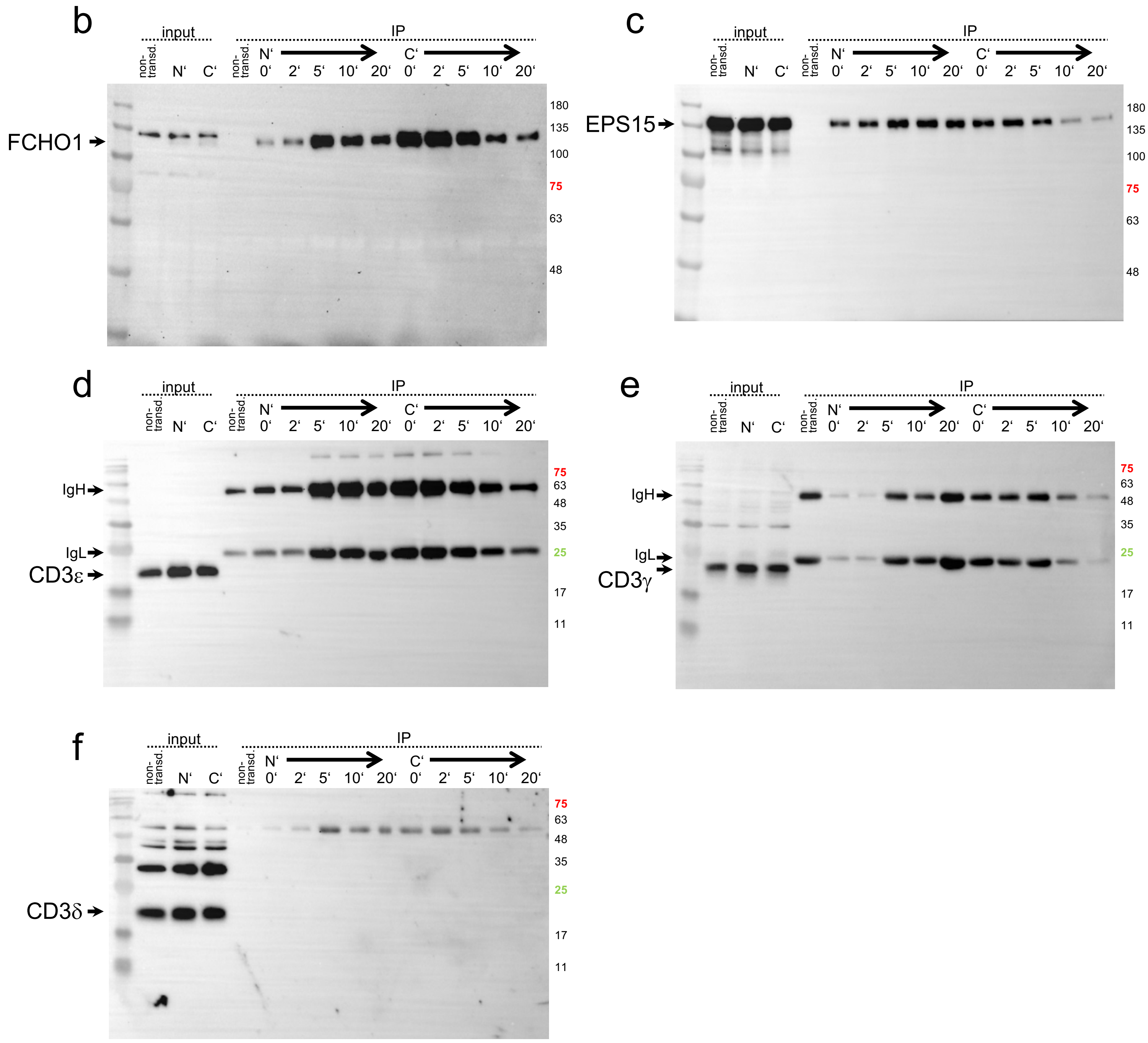

Supplementary Figure 9. FCHO1 does not bind to CD3 ε, g, and d subunits of the TCR complex. Immunoprecipitation experiments with lysates prepared from FCHO1-deficient Jurkat cells stably overexpressing N' or C'-Flag wt FCHo1 fusion protein. Prior to immunoprecipitation cells were starved for a minimum 45' at 37°C and subsequently stimulated as indicated with anti-CD3 Ab to induce clustering of TCR complexes. Non-transduced Jurkat FCHO1-deficient cells served as negative control. (a) Amino acid sequence of CD3 ε, d and d. Cytoplasmic motif is marked in green, DxY or DxxY putative sorting motifs in red. (b) FCHO1 and (c) EPS15 served as positive immunoprecipitation controls. (d-f) Western blots of CD3 ε, d and d components of the TCR complex. The molecular mass of proteins is shown in kDa. Specific bands as well as Ig heavy (IgH) and Ig light (IgL) chains are indicated with arrows. Representative data of two independent experiments are shown.

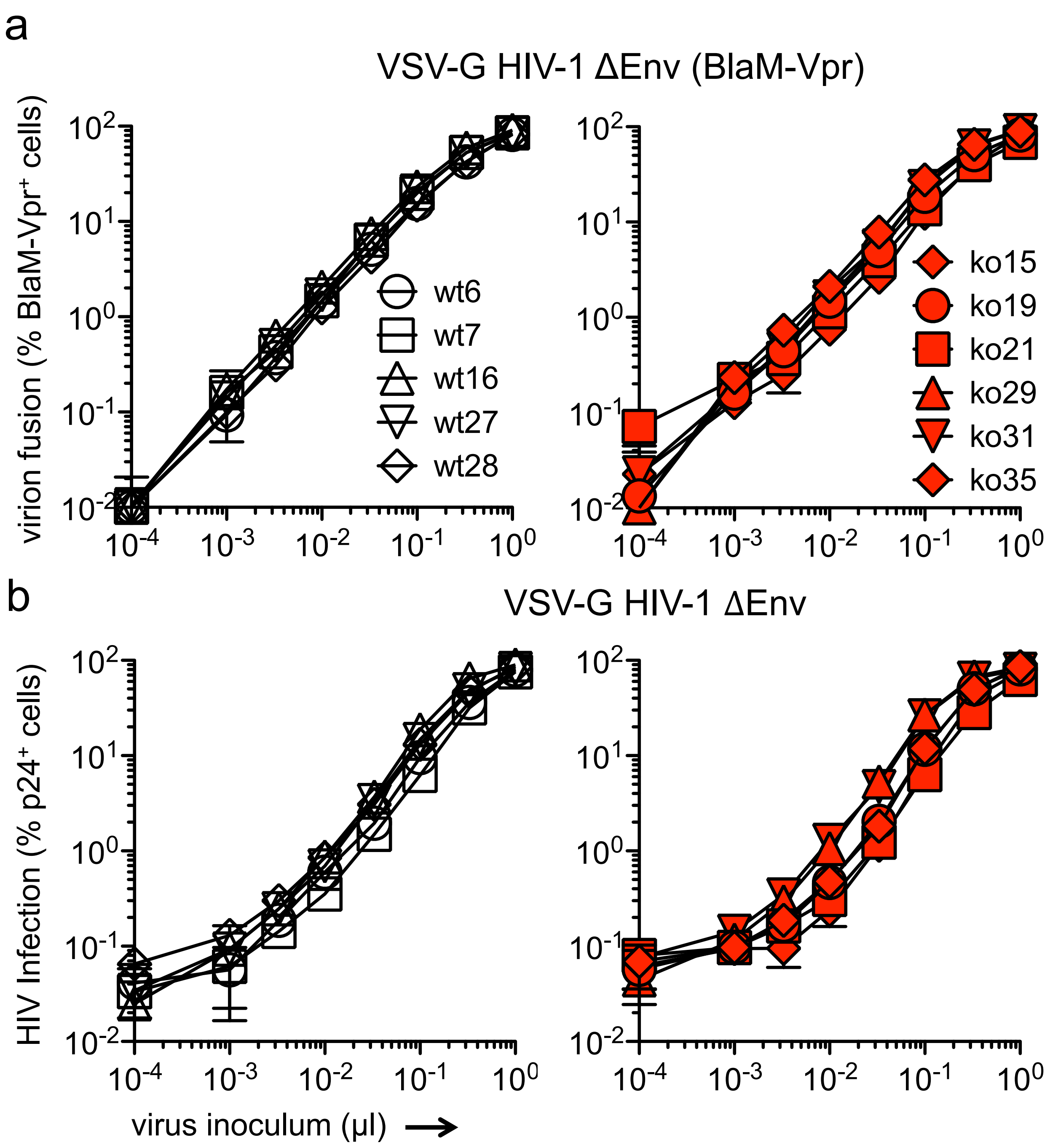

Supplementary Figure 10. FCHO1 ko in Jurkat T cells does not affect fusion and infection by HIV-1ΔEnv pseudotyped with VSV-G. (a) Virion fusion of VSV-G HIV-1ΔEnv (BlaM-Vpr) or (b) infection by VSV-G HIV-1ΔEnv of five Jurkat wt and six FCHO1 ko clones. (a, b) Jurkat wt and FCHO1 ko clones were challenged with increasing volumes of the indicated VSV-G-pseudotyped HIV-1ΔEnv. Virion fusion was monitored by flow cytometry and the percentage of cleaved CCF2+/BlaM-Vpr+ cells is plotted relative to the virus inoculum. Infection of VSV-G HIV-1 ΔEnv was monitored by intracellular HIV-1 p24 staining two days post challenge and the relative percentage of p24-positive cells is plotted relative to the virus inoculum. Values represent the arithmetic means of technical triplicates.

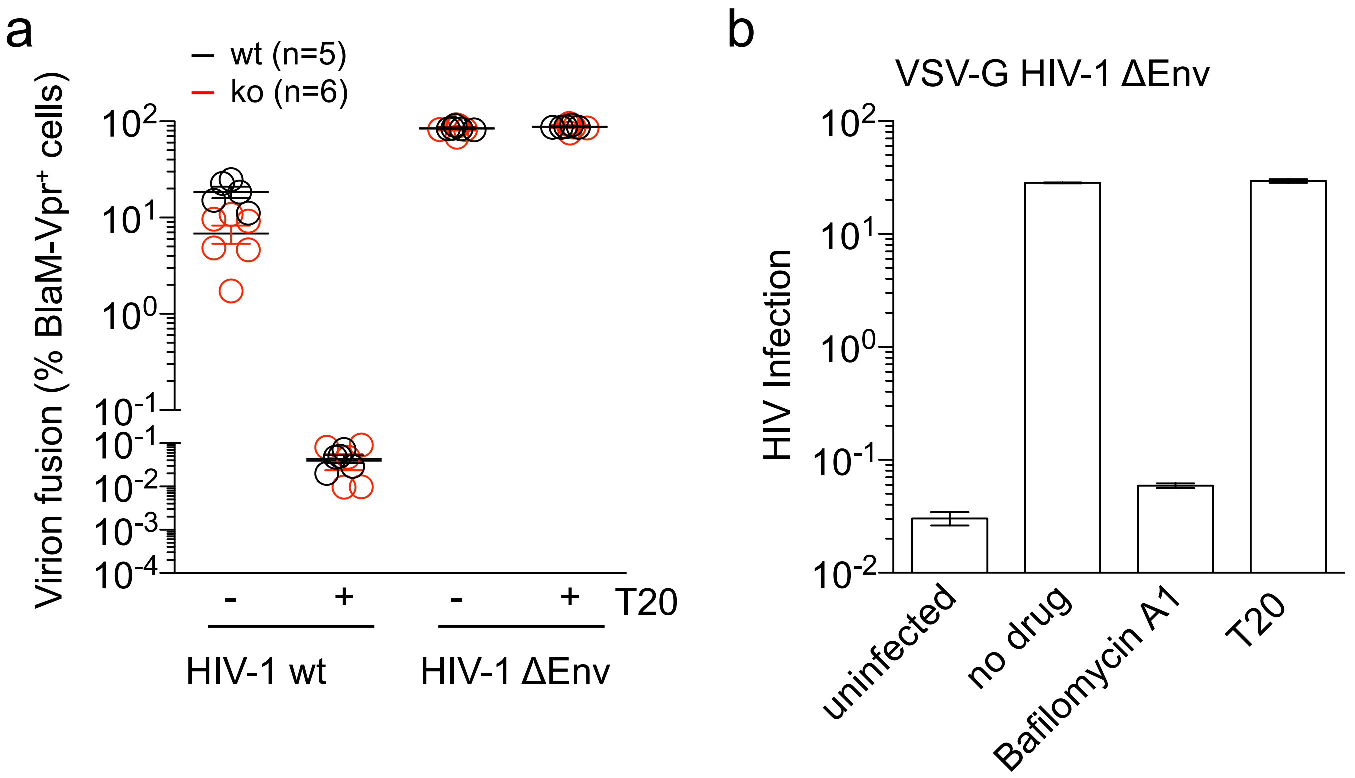

Supplementary Figure 11. **CME-dependent infection with VSV-G-pseudotyped HIV-1 is inhibited by bafilomycin A1, but not by the lack of FCHO1.** (a) Jurkat wt and FCHO1 ko clones were pre-incubated for 1 h either with the HIV-1-specific fusion inhibitor T20 or PBS and then challenged with HIV-1wt (BlaM-Vpr) or VSV-G HIV-1ΔEnv (BlaM-Vpr). Virion fusion was monitored by flow cytometry and the relative percentage of cleaved $CCF2^{+}$/BlaM-Vpr^{+} cells is given. The symbols depicted represent the arithmetic means + standard deviations of technical triplicates from one representative experiment for five Jurkat wt clones (red circles) or six FCHO1 ko clones (grey squares). (b) Jurkat T cells were pre-treated with either bafilomycin A1, T20 or PBS (no drug) and subsequently challenged with VSV-G HIV-1ΔEnv. Two days later, cells were analyzed for the percentage of HIV-1 p24-positive cells by flow cytometry. Uninfected cells served as a reference. Histogram bars represent the arithmetic mean + standard deviation of three technical replicates from one representative experiment.

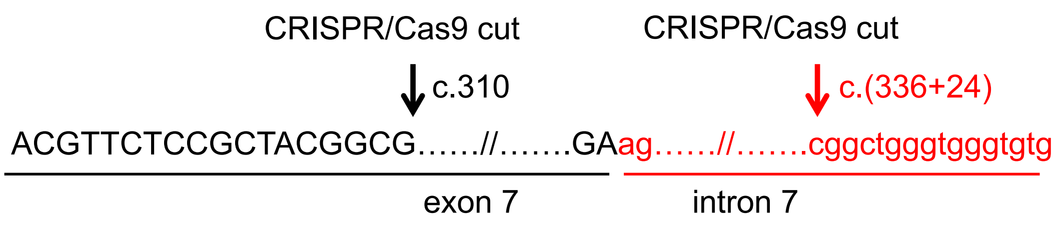

expected sequence after cut: ACGTTCTCCGCTACGGCGcggctgggtgggtgtg (exon 7 | intron 7)

Clone J15
allele 1: ACGTTCTCCGCTACG*CGA*cggctgggtgggtgtg
allele 2: ACGTTCTCCGCTACG*GA*cggctgggtgggtgtg

Clone J19
allele 1: ACGTTCTCCGCTACG*CCC*cggctgggtgggtgtg
allele 2: ACGTTCTCCGCTAC*CC*cggctgggtgggtgtg

Clone J21
allele 1: ACGTTCTCCGCTACG*CG*cggctgggtgggtgtg
allele 2: ACGTTCTCCGCTACcggctgggtgggtgtg

Clone J29
allele 1: ACGTTCTCCGCTAC*NNNN*cggctgggtgggtgtg
allele 2: ACGTTCTCCGCTACG*N*cggctgggtgggtgtg

Clone J35
allele 1: ACGTTCTCCGCTAC*GG*cggctgggtgggtgtg
allele 2: ACGTTCTCCGCTACGcggctgggtgggtgtg

Supplementary Figure 12. Examples of CRISPR/Cas9-mediated deletion damaging FCHO1 locus at exon7 – intron 7 junction. Deletion at an intron-exon junction ensures aberrant splicing of the pre-mRNA transcript, whereas the deletion at an exon distant from the beginning of the protein prevents from usage of an alternative start codon.

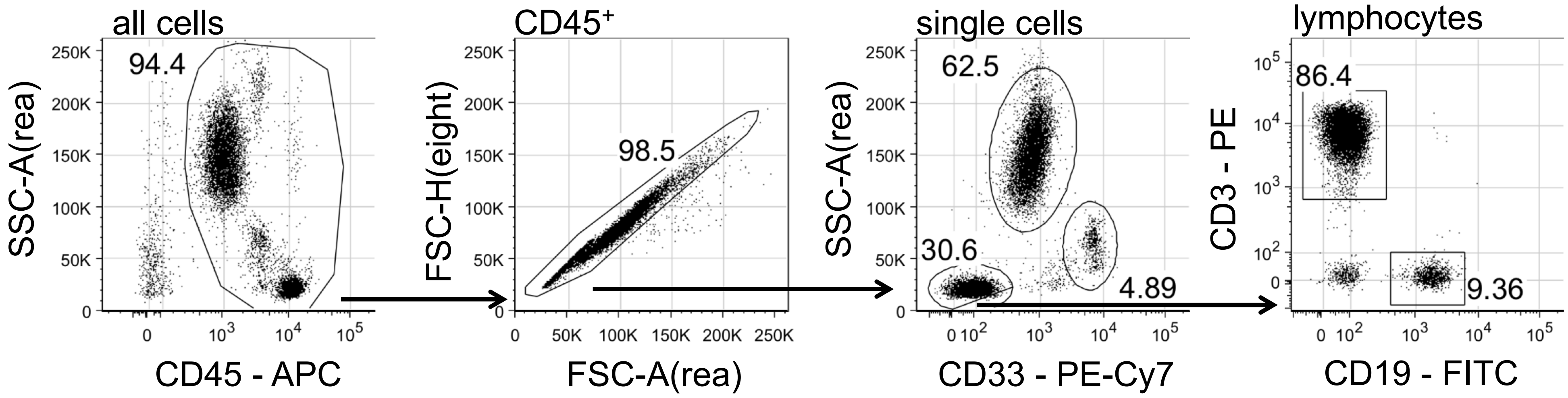

Supplementary Figure 13. Example of gating strategy to assess frequency of leukocytes in human peripheral blood. CD45 staining was used to discriminate between leukocytes and remaining erythrocytes and platelets. FSC-A and FSC-H parameters were used to exclude doublets.

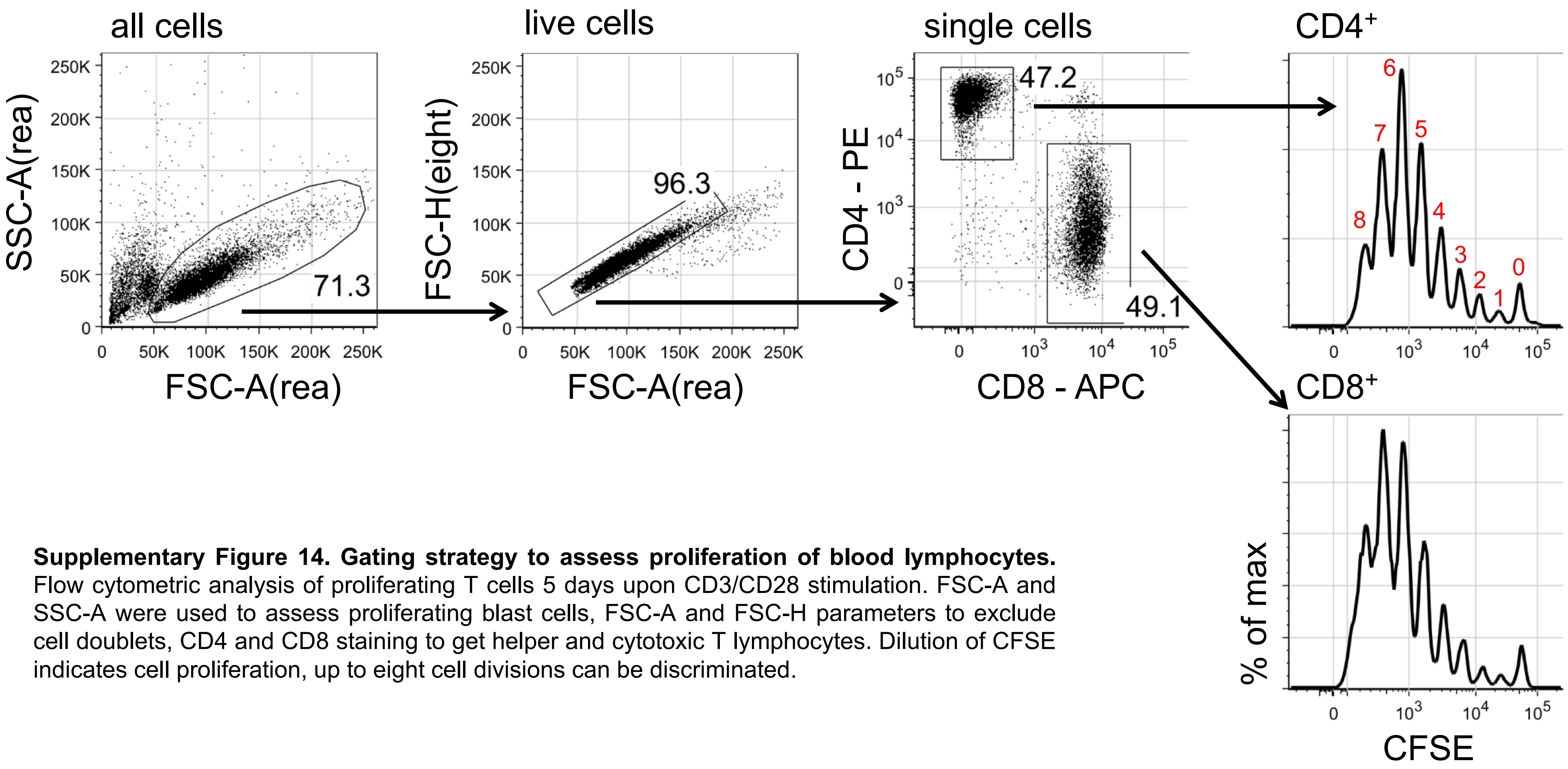

Supplementary Figure 14. Gating strategy to assess proliferation of blood lymphocytes. Flow cytometric analysis of proliferating T cells 5 days upon CD3/CD28 stimulation. FSC-A and SSC-A were used to assess proliferating blast cells, FSC-A and FSC-H parameters to exclude cell doublets, CD4 and CD8 staining to get helper and cytotoxic T lymphocytes. Dilution of CFSE indicates cell proliferation, up to eight cell divisions can be discriminated.

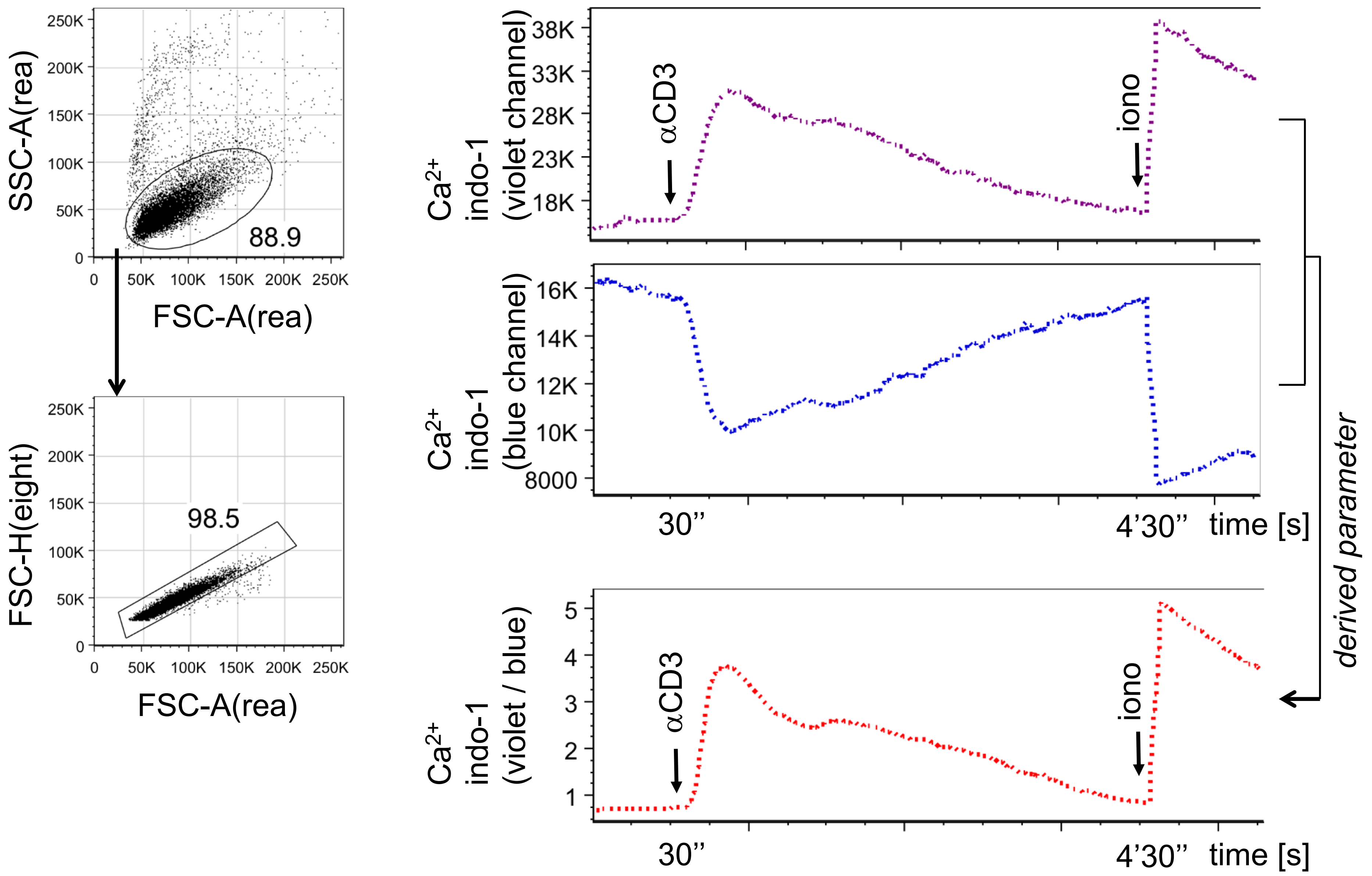

Supplementary Figure 15. Flow cytometry work flow to assess Ca^{2+} release from ER upon CD3 stimulation. Prior to CD3 stimulation Jurkat cells were loaded with an indo-1, Ca^{2+}-sensitive dye. Changes in cytoplasmic concentration of Ca^{2+} can be followed due to the fact the emission of indo-1 shifts from about 475 nm without Ca^{2+} ("blue" channel) to about 400 nm ("violet" channel) with Ca^{2+} when excited at about 350 nm. Usage of 400/475 ratio parameter (red line) reduces the effect of uneven dye loading between the samples, uneven size of the cells, photobleaching etc.

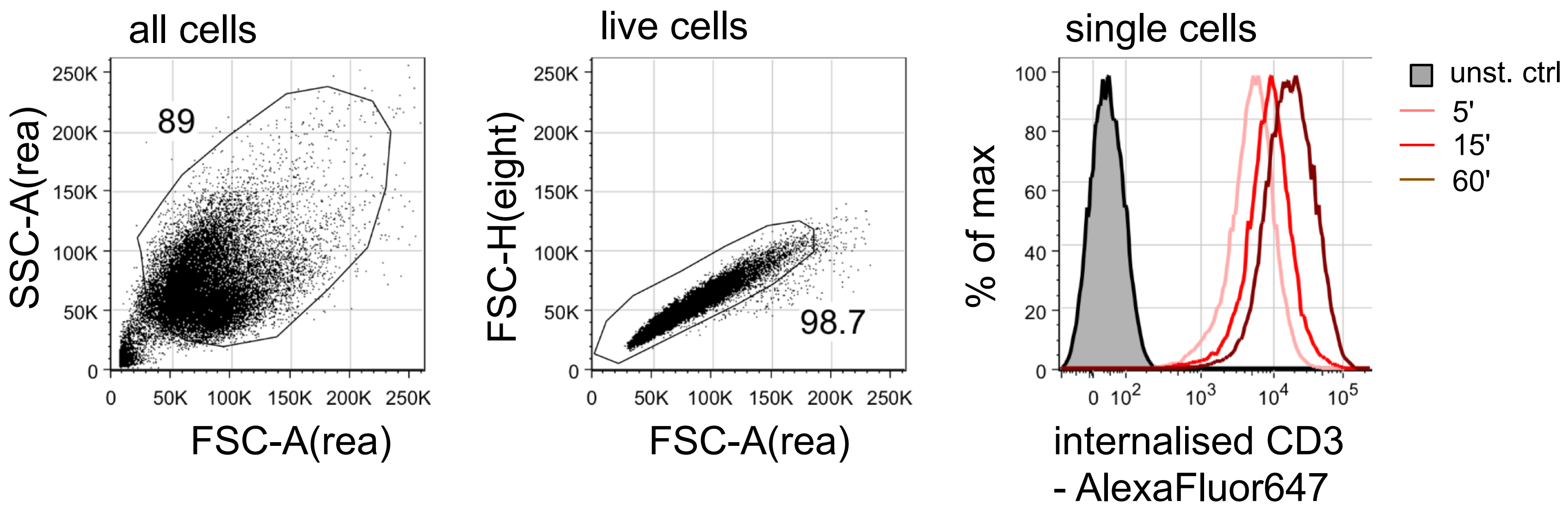

Supplementary Figure 16. Gating strategy to assess TCR-internalisation. FSC-A and SSC-A parameters were used to discriminate between cells and debris, FSC-A and FSC-H parameters to exclude cell doublets. Internalisation of TCR was followed over time after striping of remaining surface TCR.

	A1	B1	C1	D1	E1	E2	E3	F1	F2	G1
blood cells count										
White blood cells (WBC)	2400/µl	2400/µl [4800-10800/µl]	3470/µl [4800-10800/µl]	6640/µl [4800-10800/µl]	2920	NA	4360/µl	7200/µl [5200-11000]	8600/µl [5200-11000]	3890/µl [5500-14000/µl]
Neutrophils	55%	83.5% [43-65%]	73.2% [43-65%]	64.8% [43-65%]	61% [40-67%]	NA	42% [40-67%]	74% [45.5-73.1]	62% [0.14-1.59]	40% [30-53%]
Lymphocytes	8.3%	10.3% [20.5-45.5%]	15.9% [20.5-45.5%]	21.5% [20.5-45.5%]	18.6% [33-60]	NA	46% [33-60]	14% [22.3-49.9]	16% [22.3-49.9]	27% [37-52%]
Monocytes	10%	3.8% [5.5-11.7%]	6.3% [5.5-11.7%]	9.6% [5.5-11.7%]	10% [4.7-12.5%]	NA	8.8% [4.7-12.5%]	2% [0.7-7.5]	12% [0.7-7.5]	7.3% [0-5%]
Eosinophils	26.3%	0.3% [0.9-2.9%]			5% [0-7%]	NA	3% [0-7%]	10% [0.0-4.4]	10% [0.0-4.4]	1.6% [0-4%]
Basophils	0.4%	0.3% [0.2-1%]			1.9 [0-1.5%]	NA	0.3%[0-1.5%]	0% [0.2-1.2]	0% [0.2-1.2]	0.3% [0-1%]
lymphocytes										
$CD3^+$	58/µl (30%)	151/µl (61.3%) [60-85%]	305/µl (55.3%) [60-85%]	917/µl (64.1%) [60-85%]	489 (90%) [60-85%]	NA	891/µl (65%) [60-85%]	554/µl [1400-3700]	247/µl [1400-3700]	1164/µl (65.4%)
$CD4^+$	17/µl (9%)	53/µl (21.6%) [29-59%]	46/µl (8.3%) [29-59%]	177/µl (12.4%) [29-59%]	81 (15%) [36-63%]	NA	137/µl (10%) [36-63%]	100/µl [700-2200]	178/µl [700-2200]	78/µl (4.4%)
$CD8^+$	56/µl (29%)	104/µl (42.2%) [19-48%]	247/µl (44.9%) [19-48%]	532/µl (37.2%) [19-48%]	396 (73%) [15-40%]	NA	754/µl (55%) [15-40%]	443/µl [490-1300]	68/µl [490-1300]	981/µl (55%)
$CD19^+$	31/µl (16%)	42/µl (17.1%) [11-16%]	67/µl (12.2%) [11-16%]	256/µl (17.9%) [11-16%]	0 (0%) [5-25%]	NA	625/µl (31%) [5-25%]	372/µl [390-1400]	756/µl [390-1400]	290/µl (16%) [7-23%]
$CD16^+$	88/µl (46%)	54/µl (22%) [5-20%]	164/µl (29.8%) [5-20%]	237/µl (16.6%) [5-20%]	105 (20%) [5-20%]	NA	400/µl (25%) [5-20%]	40/µl [100-600]	247/µl [100-600]	279/µl (15%)
immunoglobulins										
IgG	3.76 g/l [5.7 – 15.5 g/l] substituted	529 mg/dL [740-1450 mg/dL] substituted	471 mg/dL [700-1630 mg/dL] substituted	581 mg/dL [700-1630 mg/dL] substituted	420 mg/dL [470-1230 mg/dL] substituted	NA	316 mg/dL [470-1230 mg/dL] substituted	8.5 g/L Substituted [3.45-12.36]	9.0 g/L Substituted [3.45-12.36]	1400 mg/dL [500-1300 mg/dL]
IgM	0.25 g/l [0.6 – 3.0 g/l]	137 mg/dL [76-195 mg/dL]	17.4 mg/dL [65-206 mg/dL]	59.3 mg/dL [65-206 mg/dL]	20 mg/dL [45-169 mg/dL]	NA	114 mg/dL [45-169 mg/dL]	0.23 g/L [0.43-2.07]	0.36 g/L [0.43-2.07]	700 mg/dL [400-1800 mg/dL]
IgA	0.77 g/l [0.5 – 2.3 g/l]	89.6 mg/dL [80-190 mg/dL]	9.77 mg/dL [73-187 mg/dL]	14.1 mg/dL [73-187 mg/dL]	0 mg/dL [21-145 mg/dL]	NA	49 mg/dL [21-145 mg/dL]	0.57 g/L [0.14-1.59]	0.13 g/L [0.14-1.59]	25mg/dL [40-180 mg/dL]

Supplementary Table 1. Clinical laboratory investigations for patients A1-G1. Presented data were collected during initial admission of patients to the wards and before starting any treatment. Data in the square brackets indicate normal values for respective measurement. Frequencies of lymphocytes in the brackets were calculated in the reference to $CD45^+$ cells.

patient	chromosome	position	reference allele	alternative allele	gene	cDNA (ENST00000594202)	protein
A1	19	17893924	G	C	FCHO1	c.2036G>C	p.Arg679Pro
B1	19	17873643	G	C	FCHO1	c.100G>C	p.Ala34Pro
C1 & D1	19	17893910	T	TG	FCHO1	c.2023insG	p.Val625GlyfsTer13
E1, E2, E3	19	17881387	G	A	FCHO1	c.489+1G>A	*unknown, IVS8 splice donor*
F1, F2	19	17877476	A	C	FCHO1	c.195-2A>C	*unkown, IVS6 splice acceptor*
G1	19	17893836	C	T	FCHO1	c.1948C>T	p.Arg650Ter

Mutation Significance Cutoff (MSC) Scores (http://pec630.rockefeller.edu:8080/MSC/)

patient	CADD Score	MSC-CADD score	MSC-CADD impact prediction	PolyPhen2 score	PolyPhen2 prediction	MSC-PolyPhen2 Score	MSC-PolyPhen2 impact prediction
A1	32.000	5.744	high	0.999	probably damaging	0.239	high
B1	28.700	5.744	high	1.000	probably damaging	0.239	high
C1 & D1	35.000	5.744	high	NA	NA	0.239	NA
E1, E2, E3	25.700	5.744	high	NA	NA	0.239	NA
F1, F2	24.500	5.744	high	NA	NA	0.239	NA
G1	43.000	5.744	high	NA	NA	0.239	NA

Supplementary Table 2. Chromosomal localization of all identified genetic variants (upper panel) and predicted influence of those mutations on protein function (bottom panel).

Western Blot

antigen	dilution	source	catalog number
beta-actin	1:1000	Santa Cruz	sc-47778 HRP
FCHO1	1:1000	Thermo Fisher Scientific	PA5-31603, polyclonal
FCHO1	1:1000	Abcam	ab84740, polyclonal
EPS15	1:2000	Cell Signaling	12460S, clone D3K8R
EPS15R	1:1000	Abcam	ab76004, clone EP1146Y
Adaptin	1 :1000	Abcam	ab2730, clone AP6
CD3 epsilon	1:1000	Cell Signaling	4443S, clone CD3-12
CD3 delta	1:500	Thermo Fisher Scientific	PA5-28313, polyclonal
CD3 gamma	1:500	Thermo Fisher Scientific	PA5-29387, polyclonal
GAPDH	1:1000	Santa Cruz	sc-32233, clone 6C5
anti-mouse HRP	1:2000	BD Biosciences	554002, polyclonal
anti-rabbit HRP	1:2000	Cell Signaling	7074S, polyclonal
anti-rat HRP	1:2000	Cell Signaling	7077S, polyclonal

flow cytometry

antigen	conjugate	dilution	source	catalog number
CD45	BV711	1:50	BD Biosciences	564358, clone HI30
CD45	APC	1:50	BioLegend	304012, clone HI30
CD33	PE-Cy7	1:100	BioLegend	366618, clone P67.6
CD3	PE	1:200	BioLegend	300308, clone HIT3a
CD19	FITC	1:50	BioLegend	302206, clone HIB19
CD8a	APC	1:50	BD Biosciences	555369, clone RPA-T8
CD4	PE-Cy7	1:50	BioLegend	357410, clone A161A1
CD45	APC	1:50	BioLegend	304012, clone HI30

Supplementary Table 3. List of antibodies used in Western Blot and FACS.
