# Supplementary material for: Human FCHO1 deficiency reveals role for clathrin-mediated endocytosis in development and function of T cells
Source: Nat Commun. 2020 Feb 25;11:1031. doi: 10.1038/s41467-020-14809-9 (PMC7042371; doi:10.1038/s41467-020-14809-9)
Supplement: Supplementary file 5 — Reporting Summary [file 41467_2020_14809_MOESM5_ESM.pdf]

nature research

Corresponding author(s): Prof. Christoph Klein
Dr Marcin Lyszkiewicz

Last updated by author(s): 2018/12/10

Reporting Summary

Nature Research wishes to improve the reproducibility of the work that we publish. This form provides structure for consistency and transparency in reporting. For further information on Nature Research policies, see Authors & Referees and the Editorial Policy Checklist.

Statistics

For all statistical analyses, confirm that the following items are present in the figure legend, table legend, main text, or Methods section.

n/a	Confirmed	
☐	☒	The exact sample size (*n*) for each experimental group/condition, given as a discrete number and unit of measurement
☐	☒	A statement on whether measurements were taken from distinct samples or whether the same sample was measured repeatedly
☐	☒	The statistical test(s) used AND whether they are one- or two-sided<br>*Only common tests should be described solely by name; describe more complex techniques in the Methods section.*
☐	☒	A description of all covariates tested
☐	☒	A description of any assumptions or corrections, such as tests of normality and adjustment for multiple comparisons
☐	☒	A full description of the statistical parameters including central tendency (e.g. means) or other basic estimates (e.g. regression coefficient) AND variation (e.g. standard deviation) or associated estimates of uncertainty (e.g. confidence intervals)
☐	☒	For null hypothesis testing, the test statistic (e.g. *F*, *t*, *r*) with confidence intervals, effect sizes, degrees of freedom and *P* value noted<br>*Give P values as exact values whenever suitable.*
☒	☐	For Bayesian analysis, information on the choice of priors and Markov chain Monte Carlo settings
☐	☒	For hierarchical and complex designs, identification of the appropriate level for tests and full reporting of outcomes
☒	☐	Estimates of effect sizes (e.g. Cohen's *d*, Pearson's *r*), indicating how they were calculated

Our web collection on statistics for biologists contains articles on many of the points above.

Software and code

Policy information about availability of computer code

Data collection	For data collection following software was used: whole-exome sequencing (WES) - BWA software, version 0.7.15; flow cytometry data - FACS diva software (BD); confocal microscopy - Zen Blue software, version 2.0x (Zeiss); Western Blot collection - Image Lab software (Bio-Rad laboratories).
Data analysis	For data analysis following software was used: whole-exome sequencing (WES) - BWA software (version 0.7.15) for short sequence alignment, GATK pipeline (version 3.6) to call the variants and recalibrate, VEP release 85 to annotate the final variants, effects of filtered variants on protein were predicted with SIFT and PolyPhen2; crystal structure of FCHO1 domains were modeled using PyMol software with mutalyzer wizard; Western Blot pictures - Quantity One or Image Lab software (bith Bio-rad laboratories); confocal microscopy pictures and movies - Zen Blue software, version 2.0x (Zeiss); flow cytometry data - FlowJo Software v.8 or 9 (TreeStar); statistical analysis - GraphPadPrism software v.6; to assess Pearson correlaion and colocalization coefficient on selected cell fragments Zen Blue software, version 2.0x

For manuscripts utilizing custom algorithms or software that are central to the research but not yet described in published literature, software must be made available to editors/reviewers. We strongly encourage code deposition in a community repository (e.g. GitHub). See the Nature Research guidelines for submitting code & software for further information.

Data

Policy information about availability of data

All manuscripts must include a data availability statement. This statement should provide the following information, where applicable:

- Accession codes, unique identifiers, or web links for publicly available datasets
- A list of figures that have associated raw data
- A description of any restrictions on data availability

The identified FCHO1 mutations have been submitted to the ClinVar database with accession numbers XXX. According to current regulatory frameworks, exome sequencing data cannot be made publicly available.

Field-specific reporting

Please select the one below that is the best fit for your research. If you are not sure, read the appropriate sections before making your selection.

☒ Life sciences ☐ Behavioural & social sciences ☐ Ecological, evolutionary & environmental sciences

For a reference copy of the document with all sections, see nature.com/documents/nr-reporting-summary-flat.pdf

Life sciences study design

All studies must disclose on these points even when the disclosure is negative.

Sample size	Sample size was determined by number of individuals carrying the rare FCHO1 variants. All individuals who were identified to carry rare homozygous FCHO1 variants are included in manuscript.<br>Quantification of Pearson correlation or colocalization coefficient of FCHO1 wild-type and all tested mutants with EPS15, adaptin and clathrin was preformed on pooled data of two to three independent experiments. Each symbol represents one region of 25µm2. Up to three regions per cells were quantified, minimum 50 data points were collected for each FCHO1 variant.<br>For statistical analysis of the confocal microscopy pictures minimum 50 measures per each FCHO1 variant were collected. Data are pooled of two to three independent experiments.<br>Pearson correlation of FCHO1 and clathrin from individual movies are pooled data from three independent experiments. Each symbol represents one square region of 25µm2. Up to three regions per cells were quantified, minimum 50 regions were assessed.
Data exclusions	For confocal microscopy analysis only cell with low to moderate expression of FCHO1 were used, as high expression of FCHO1 may results in dominant-negative effects on CCP dynamic. For analysis of fixed samples, cells which partially detached from cover slip or appeared in anyway damaged were excluded from analysis. Those criteria were set before the experiment started. Otherwise there was not data exclusion.
Replication	In order to assure reproducibility, following measures were taken.<br>1/ To minimize the risk of accidental mutagenesis introduced during CRISPR/Cas9 modification and subsequent (sub)clonal selection, minimum five independent (sub)clones of cells sufficient or deficient for FCHO1 were taken for analysis. Whenever possible (i.e. Ca2+ release or TCR internalization assay performed on Jurkat cells) multiple (from three to five) clones of both wt and ko genotype were analyzed in one experiment. For confocal microscopy assessments different clones of SK-MEL cells deficient for FCHO1 were used interchangeably.<br>2/ Pearson correlation and collocalization coefficient of confocal microscopy images and movies was performed on data obtained from minimum two (mostly three) independent experiments. Details how many replicates were used in particular experiment are depicted in figure legends.<br>3/ Due to limited access to confocal microscope, initial experiments were performed on fixed cells stably transduced with GFP-FCHO1 variants in Zeiss Laboratories (Munich). Further, all fix-cell experiments were repeated on transiently tranfected cells in house (LMU, Munich). Result obtained from stably and transiently transduced cells were essentially identical.<br>4/ Confocal microscopy experiments were prepared and measured independently by M. Ł., N. Z. and L. F. with similar results.<br>5/ Western blot experiments were performed independently by M. Ł. and L. F. with similar results.
Randomization	For assays, where mild change of the experimental conditions might result in profound effects samples were measured alternately. For instance, Ca2+ release assay - multiple Jurkat clones of wt and ko genotype as well as ko reconstituted with indicated FCHO1 variants were measured alternately.
Blinding	For assessment of confocal microscopy images blinding was not possible, as the effects of tested FCHO1 variants were obvious even for researchers not involved in the studies.

Reporting for specific materials, systems and methods

We require information from authors about some types of materials, experimental systems and methods used in many studies. Here, indicate whether each material, system or method listed is relevant to your study. If you are not sure if a list item applies to your research, read the appropriate section before selecting a response.

Materials & experimental systems

n/a	Involved in the study	
☐	☒	Antibodies
☐	☒	Eukaryotic cell lines
☒	☐	Palaeontology
☒	☐	Animals and other organisms
☐	☒	Human research participants
☒	☐	Clinical data

Methods

n/a	Involved in the study	
☒	☐	ChIP-seq
☐	☒	Flow cytometry
☒	☐	MRI-based neuroimaging

Antibodies

Antibodies used

Following primary antibodies were used for Western blot analysis: FCHO1–rabbit, polyclonal, PA5-31603, lot Q12081994A, Thermo Scientific or rabbit, polyclonal, 84740, lot GR214150-4, Abcam; EPS15–rabbit, clone D3K8R, Cell Signalling; EPS15R–rabbit, clone EP1146Y, Abcam; adaptin–mouse, clone AP6, Abcam; CD3epsilon–rat, clone OKT3, Thermo Fisher Scientific; CD3delta–rabbit, polyclonal, Thermo Fisher Scientific; CD3 gamma–rabbit, polyclonal, Thermo Fisher Scientific; GAPDH–mouse, clone 6C5, Santa Cruz. Secondary Ab: α-mouse (BD), α-goat (Santa Cruz), or α-rabbit (CST) conjugated to horseradish peroxidase. Following primary Ab were used in confocal microscopy: rabbit-α-Eps15 (clone D3K8R, Cell Signalling) or mouse-α-Adaptin (AP6, Abcam). Following secondary antibodies coupled to fluorochromes were used: goat-α-rabbit IgG, AlexaFluor 405 (Invitrogen) and goat-α-mouse IgG, AlexaFluor 633 (Invitrogen).
For FACS analysis following Ab were used: α-CD45 BV711 (HI30), α-CD33 PE-Cy7 (P67.6), α-CD3 PE (HIT3a), α-CD19 FITC (HIB19), α-CD8α APC (RPA-T8), α-CD4 PE-Cy7 (A161A1) all from Biolegend and α-CD3 (OKT3) BD.

Validation

Specificity of polyclonal Ab targeted against human FCHO1 eptiopes were tested in Western blot using both positive and negative controls. Cells transfected with WT FCHO1 construct served as positive control whereas cells with genetically disrupted FCHO1 locus (CRISPR/Cas/9 mediated modification were resulting in frame-shitf mutations in both chromosomes) were used as negative control. Specificity of other antibodies used in WB were controlled using protein size "ladder" and by comparison of band pattern with those provided by manufacturer and other publications.
For confocal microscopy experiments only the Ab which give minimal unspecific background in WB analysis were used.
All antibodies used for flow cytometric analysis were monoclonal, provided by well-established manufacturers and staining pattern obtained from healthy volunteers was as expected.

Eukaryotic cell lines

Policy information about cell lines

Cell line source(s)

The SK-MEL-2 cell line, engineered using zinc finger nucleases (ZNF) genome editing to stably express RFP under the endogenous human clathrin light chain A locus (CLTA-RFP), was kindly provided by David G. Drubin, University of Berkeley. Jurkat cells were purchased from ATCC. HEK293T cells and NIH-3T3 cells were obtained from DSMZ–German Collection of Microorganisms and Cell Cultures. Patient fobroblasts and EBV-LCL cells were derived from patients or healthy family members.

Authentication

SK-MEL-2 cells were, as expected, expressing RFP signal from endogenous locus. ATTC and DSMZ are reliable cell line providers, those cells were not further validated.

Mycoplasma contamination

All cell lines were routinely tested for mycoplasma and were mycoplasma negative throughout the study.

Commonly misidentified lines
(See ICLAC register)

N.A.

Human research participants

Policy information about studies involving human research participants

Population characteristics

All relevant information is provided in Table 1 and Sup Table 1

Recruitment

Patients were referred to the clinical and scientific team of Professor Christoph Klein for further investigations.

Ethics oversight

Informed consent/assent was obtained from all legal representatives and patients. Genetic and functional studies on biosamples from patients and their relatives were performed under the framework of a scientific project entitled “Genetic characterization of congenital bone marrow failure and immunodeficiency syndromes”. This study has been approved in 2011 by the ethics committee at LMU (438-11).

Note that full information on the approval of the study protocol must also be provided in the manuscript.

Flow Cytometry

Plots

Confirm that:

☐ The axis labels state the marker and fluorochrome used (e.g. CD4-FITC).

☒ The axis scales are clearly visible. Include numbers along axes only for bottom left plot of group (a 'group' is an analysis of identical markers).

☒ All plots are contour plots with outliers or pseudocolor plots.

☒ A numerical value for number of cells or percentage (with statistics) is provided.

Methodology

Sample preparation

1/ Assessment of lymphocyte frequency in peripheral blood. Blood samples were washed with PBS and stained with the

Sample preparation	following antibodies for 20min at RT: α-CD45 BV711 (HI30), α-CD33 PE-Cy7 (P67.6), α-CD3 PE (HIT3a), α-CD19 FITC (HIB19), α-CD8α APC (RPA-T8), α-CD4 PE-Cy7 (A161A1) all from Biolegend. Red blood cells were lysed using 1×BD FACS Lysing Solution (BD Biosciences) according to the manufacturer's instructions.<br>2/ Assessment of intracellular calcium flux. Up to five different FCHo1-sufficient or -deficient Jurkat clones were incubated for 1 h in Ca2+ and Mg2+ -free Dulbecco's serum-free medium (Invitrogen) at room temperature at a density of 107 cells/mL. Cells were then loaded with Fluo-4 (3 µM) and FuraRed (6 µM) for 45 min at 37oC. Further, cells were rested for 30 to 45 min at 37oC. After establishing of a baseline for 30 s, cells were stimulated with α-CD3 antibody (OKT3, 1mg/ml, BD Biosciences) and goat-α-mouse polyclonal antibody (0.5mg/ml, Jackson ImmunoResearch) to allow cross-linking and data acquisition was continued for 4 additional minutes. To ensure cell viability, one minute before the end of acquisition 2 µg/mL ionomycin (Sigma) was added as positive Ca2+-flux control.<br>3/ TCR internalisation. FCHO1-sufficient or -deficient Jurkat cells were stained with α-CD3 Ab (OKT3) in cold and TCR internalisation was assessed over time at 37oC in the presence of anti-mouse F(ab')2 fragments labelled with Ax647. After 2, 5, 15, 45 and 60 minutes of stimulation the remaining surface TCRs were stripped, thus fluorescent signal corresponds only to the internalised TCR.
Instrument	LSRFortessa (BD Bioscience)
Software	FACS Aria software was used to collect the data, FlowJo v.8.x or 9.x was used to analayse the data
Cell population abundance	N.A.
Gating strategy	Essential gating strategy is shown on Figure 5. FSC-A(rea) and FCS-H(eight) parameters were used to discriminate singlets from doublets. Further, lymphocytes were defined as CD45 hi CD33- SSC lo, neutrophiles as CD45 lo CD33 int SSC hi, monocytes as CD45 lo CD33 hi SSC lo-int. Among lymphocytes CD3+ T cells and CD19+ B cells can be distinguished. Further, T cell can be subdivided into CD4+ helper and CD8+ cytotoxic T cells.

☐ Tick this box to confirm that a figure exemplifying the gating strategy is provided in the Supplementary Information.
