# Supplementary material for: Human FCHO1 deficiency reveals role for clathrin-mediated endocytosis in development and function of T cells
Source: Nat Commun. 2020 Feb 25;11:1031. doi: 10.1038/s41467-020-14809-9 (PMC7042371; doi:10.1038/s41467-020-14809-9)
Supplement: Supplementary file 6 — Description of Additional Supplementary Files [file 41467_2020_14809_MOESM6_ESM.pdf]

Title: Supplementary Movie 1 – FCHO1-WT-GFP clathrin-RFP
Description: FCHO1 proteins carrying patient-associated mutations fail to interact with clathrin. Engineered SK-MEL-2 cells expressing an RFP-tagged clathrin allele from endogenous locus and a GFP-FCHO1 fusion protein were imaged with 1s intervals using confocal microscopy. In the first section of each movie colours are separated, the GFP signal appears first, followed by the RFP signal. Later, both GFP and RFP signals appear combined.

Title: Supplementary Movie 2 – FCHO1-A34P-GFP clathrin-RFP
Description: FCHO1 proteins carrying patient-associated mutations fail to interact with clathrin. Engineered SK-MEL-2 cells expressing an RFP-tagged clathrin allele from endogenous locus and a GFP-FCHO1 fusion protein were imaged with 1s intervals using confocal microscopy. In the first section of each movie colours are separated, the GFP signal appears first, followed by the RFP signal. Later, both GFP and RFP signals appear combined.

Title: Supplementary Movie 3 – FCHO1-Stop687-GFP clathrin-RFP
Description: FCHO1 proteins carrying patient-associated mutations fail to interact with clathrin. Engineered SK-MEL-2 cells expressing an RFP-tagged clathrin allele from endogenous locus and a GFP-FCHO1 fusion protein were imaged with 1s intervals using confocal microscopy. In the first section of each movie colours are separated, the GFP signal appears first, followed by the RFP signal. Later, both GFP and RFP signals appear combined.
